# Complete genome sequences of two *Cressdnaviricota* viruses identified in respiratory tract samples from forest musk deer in China

**DOI:** 10.1128/mra.00632-25

**Published:** 2025-11-28

**Authors:** Qing Zhang, Xiaojie Jiang, Yuan Xi, Xiao Ma, Wen Zhang

**Affiliations:** 1 Department of Microbiology, School of Medicine, Jiangsu University 12676, Zhenjiang, Jiangsu, China; 2 Qinghai Institute of Endemic Disease Prevention and Control, Xining, China; 3 Department of Clinical Laboratory, Wuxi Blood Center, Wuxi, Jiangsu, China; DOE Joint Genome Institute, Berkeley, California, USA

**Keywords:** *Cressdnaviricota*, forest musk deer, viral metagenomics, respiratory virome

## Abstract

We identified two circular single-stranded DNA viruses from forest musk deer in China through metagenomic analysis. Phylogenetic results suggest they represent unclassified *Cressdnaviricota* lineages. This study highlights the diversity of the deer’s respiratory virome and underscores the importance of wildlife virus surveillance for conservation and public health.

## ANNOUNCEMENT

The forest musk deer (*Moschus berezovskii*) is a nationally protected Class I wildlife species in China, threatened by habitat loss and poaching (1). To investigate its respiratory virome, we performed viral metagenomic analysis on nasal swabs collected from 30 individuals in Anhui Province in 2017. The samples were pooled into three groups and processed for high-throughput sequencing. Among the viruses identified, two circular single-stranded DNA viruses were affiliated with the phylum *Cressdnaviricota*, which includes highly diverse viruses infecting a broad range of hosts (2).

Samples were collected using sterile polyester swabs and stored at –80°C. Each pool of 10 swabs was suspended in calcium- and magnesium-free DPBS, vortexed, centrifuged, and filtered through 0.45  µm membranes to eliminate residual eukaryotic cells and bacteria (3). The filtrates were treated with DNase and RNase enzymes to remove unprotected nucleic acids (4). Total viral nucleic acids were extracted using the QIAamp Viral RNA Mini Kit (Qiagen). Reverse transcription and double-stranded DNA synthesis were carried out using SuperScript IV (Invitrogen) and Klenow fragment (New England Biolabs), respectively. Sequencing libraries were prepared using the Nextera XT DNA Library Preparation Kit (Illumina) and sequenced on an Illumina MiSeq platform (250 bp paired-end reads). Reads were quality-filtered (Q10, Phred v1.0.0), and host reads were removed using Bowtie 2 (v2.3.4.1) against the *M. berezovskii* genome (GCF_022376915.1). Assembly was performed with EnsembleAssembler v1.0.0(5) and refined in Geneious Prime v2019.0.5(6). Contigs were screened for vector contamination via VecScreen (UniVec). Viral candidates were identified via DIAMOND BLASTx (v2.0.15) against a curated NVNR database and validated using NCBI Viral RefSeq v219 (7). Remote homologs were detected using vFam v1.1 and HMMER3 v3.3.2(8). Open reading frame (ORFs) were predicted in Geneious; taxonomy was assigned with MEGAN v6.21.16. Circular genomes were confirmed by overlapping reads. All tools ran with default settings.

**Fig 1:**
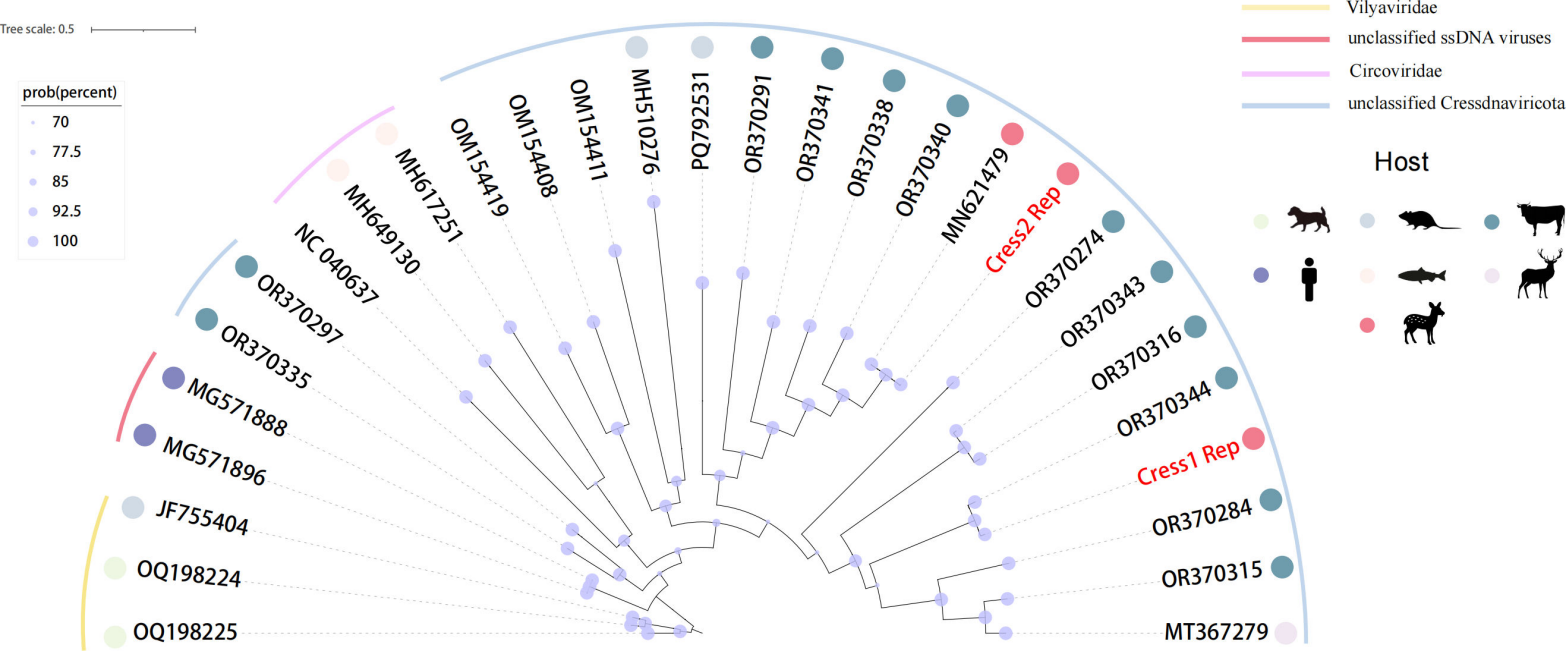
Phylogenetic analysis of Cressdnaviricota viruses. A Bayesian-inferred phylogenetic tree was constructed based on nucleic acid sequences of the Rep. Red-colored nodes indicate novel viral sequences identified in this study. We used the MUSCLE algorithm in MEGA (v11.0.13) with default parameters (9). Phylogenetic reconstruction was performed using MrBayes (v3.2.7) based on Bayesian inference with the model set to lset nst=6 rates=invgamma to account for variable substitution patterns and rate heterogeneity (10). Two independent Markov chain Monte Carlo (MCMC) runs were conducted until the average standard deviation of split frequencies dropped below 0.01, indicating convergence and robustness of the analysis (11).

The two complete viral genomes are circular, measuring 4,171 bp and 4,325 bp in length, with GC contents of 45.8% and 43.4%, respectively. Each genome contains a capsid protein ORF and a replication-associated protein (Rep) ORF, the hallmark of *Cressdnaviricota*. The average coverage depths were 19.3× and 33.1×, respectively, indicating sufficient sequencing depth to support high-confidence assembly. Cress1 clustered with a 2021 yak gut sequence from Qingdao, China (GenBank: OR370344) at 94.89% identity, while Cress2 shared 99.90% identity with a 2016 forest musk deer sequence (GenBank: MN621479). Phylogenetic analysis of Rep sequences places them in an unclassified clade within the phylum, distinct from known families. Phylogenetic analysis showed that the two newly identified *Cressdnaviricota* viruses belong to unclassified but evolutionarily distinct lineages, suggesting taxa (Fig. 1). In summary, this study identified two *Cressdnaviricota* viruses in respiratory tract samples from forest musk deer using high-throughput viromic analysis, highlighting their genomic diversity and evolutionary relationships.

## Data Availability

All raw sequence data have been deposited in the National Center for Biotechnology Information (NCBI) under BioProject PRJNA1276714 and BioSample SAMN49082469, with corresponding Sequence Read Archive (SRA) accession numbers SRR33980742–SRR33980744. The complete viral genomes are available under GenBank accession numbers PV854197 and PV854198. All data are publicly accessible without restrictions.

## References

[1] Singh PB, Saud P, Jiang Z, Zhou Z, Hu Y, Hu H. 2022. Himalayan musk deer (Moshcus leucogaster) behavior at latrine sites and their implications in conservation. Ecol Evol 12:e8772. doi:10.1002/ece3.8772 35432920 PMC9001115

[2] Krupovic M, Varsani A, Kazlauskas D, Breitbart M, Delwart E, Rosario K, Yutin N, Wolf YI, Harrach B, Zerbini FM, Dolja VV, Kuhn JH, Koonin EV. 2020. Cressdnaviricota: a virus phylum unifying seven families of rep-encoding viruses with single-stranded, circular DNA genomes. J Virol 94:e00582–20. doi:10.1128/JVI.00582-20 32269128 PMC7307096

[3] Conceição-Neto N, Zeller M, Lefrère H, De Bruyn P, Beller L, Deboutte W, Yinda CK, Lavigne R, Maes P, Van Ranst M, Heylen E, Matthijnssens J. 2015. Modular approach to customise sample preparation procedures for viral metagenomics: a reproducible protocol for virome analysis. Sci Rep 5:16532. doi:10.1038/srep16532 26559140 PMC4642273

[4] Jiang X, Liu J, Xi Y, Zhang Q, Wang Y, Zhao M, Lu X, Wu H, Shan T, Ni B, Zhang W, Ma X. 2023. Virome of high-altitude canine digestive tract and genetic characterization of novel viruses potentially threatening human health. mSphere 8:e00345–23. doi:10.1128/msphere.00345-23 37724888 PMC10597464

[5] Deng X, Naccache SN, Ng T, Federman S, Li L, Chiu CY, Delwart EL. 2015. An ensemble strategy that significantly improves de novo assembly of microbial genomes from metagenomic next-generation sequencing data. Nucleic Acids Res 43:e46–e46. doi:10.1093/nar/gkv002 25586223 PMC4402509

[6] Zhang W, Yang S, Shan T, Hou R, Liu Z, Li W, Guo L, Wang Y, Chen P, Wang X, Feng F, Wang H, Chen C, Shen Q, Zhou C, Hua X, Cui L, Deng X, Zhang Z, Qi D, Delwart E. 2017. Virome comparisons in wild-diseased and healthy captive giant pandas. Microbiome 5:90. doi:10.1186/s40168-017-0308-0 28780905 PMC5545856

[7] Liu J, Jiang X, Lei W, Xi Y, Zhang Q, Cai H, Ma X, Liu Y, Wang W, Liu N, Zhang X, Ma W, Zhao C, Ni B, Zhang W, Wang Y. 2024. Differences between the intestinal microbial communities of healthy dogs from plateau and those of plateau dogs infected with Echinococcus. Virol J 21:116. doi:10.1186/s12985-024-02364-4 38783310 PMC11112841

[8] Finn RD, Clements J, Eddy SR. 2011. HMMER web server: interactive sequence similarity searching. Nucleic Acids Res 39:W29–W37. doi:10.1093/nar/gkr367 21593126 PMC3125773

[9] Kumar S, Stecher G, Li M, Knyaz C, Tamura K. 2018. MEGA X: molecular evolutionary genetics analysis across computing platforms. Mol Biol Evol 35:1547–1549. doi:10.1093/molbev/msy096 29722887 PMC5967553

[10] Ronquist F, Teslenko M, van der Mark P, Ayres DL, Darling A, Höhna S, Larget B, Liu L, Suchard MA, Huelsenbeck JP. 2012. MrBayes 3.2: efficient Bayesian phylogenetic inference and model choice across a large model space. Syst Biol 61:539–542. doi:10.1093/sysbio/sys029 22357727 PMC3329765

[11] Shan T, Yang S, Wang H, Wang H, Zhang J, Gong G, Xiao Y, Yang J, Wang X, Lu J, et al.. 2022. Virome in the cloaca of wild and breeding birds revealed a diversity of significant viruses. Microbiome 10:60. doi:10.1186/s40168-022-01246-7 35413940 PMC9001828

